# Influence of Environmental Conditions on Tropical and Temperate Hardwood Species Bonded with Polyurethane Adhesives

**DOI:** 10.3390/ma19030589

**Published:** 2026-02-03

**Authors:** Marcin Małek, Magdalena Wasiak, Ewelina Kozikowska, Jakub Łuszczek, Cezary Strąk

**Affiliations:** 1 Institute of Civil Engineering, Faculty of Civil Engineering and Geodesy, Military University of Technology, 00-908 Warsaw, Poland; 2 Building Research Institute, Filtrowa 1, 00-611 Warsaw, Poland; m.wasiak@itb.pl (M.W.); e.kozikowska@itb.pl (E.K.); c.strak@itb.pl (C.S.); 3 Institute of Robots and Machine Design, Faculty of Mechanical Engineering, Military University of Technology, Kaliskiego 2, 00-908 Warsaw, Poland; jakub.luszczek@wat.edu.pl

**Keywords:** wooden floor, polyurethane, joints, mechanical properties

## Abstract

This research presents a comprehensive evaluation of semi-elastic polyurethane adhesives used for bonding wooden flooring, with a particular focus on both domestic (oak) and exotic hardwood species (teak, iroko, wenge, merbau). Given the increasing interest in sustainable construction practices and the growing use of diverse wood species in flooring systems, this study aimed to assess the mechanical, morphological, and surface properties of adhesive joints under both standard laboratory and thermally aged conditions. Mechanical testing was conducted according to PN-EN ISO 17178 standards and included shear and tensile strength measurements on wood–wood and wood–concrete assemblies. Specimens were evaluated in multiple aging conditions, simulating real-world application environments. Shear strength increased post-aging, with the most notable improvement observed in wenge (21.2%). Tensile strength between wooden lamellas and concrete substrates remained stable or slightly decreased (up to 18.8% in wenge), yet all values stayed above the 1 MPa minimum requirement, confirming structural reliability. Surface properties of the wood species were characterized through contact angle measurements and 3D optical roughness analysis. Teak exhibited the highest contact angle (74.9°) and the greatest surface roughness, contributing to mechanical interlocking despite its low surface energy. Oak and iroko showed high wettability and balanced roughness, supporting strong adhesion. Scanning electron microscopy (SEM) revealed stable adhesive penetration across all species and aging conditions, with no signs of delamination or interfacial failure. The study confirms the suitability of polyurethane adhesives for durable, long-lasting bonding in engineered and solid wood flooring systems, even when using extractive-rich or dimensionally sensitive tropical species. The results emphasize the critical role of surface morphology, wood anatomy, and adhesive compatibility in achieving optimal bond performance. These findings contribute to improved material selection and application strategies in flooring technology. Future research should focus on bio-based adhesive alternatives, chemical surface modification techniques, and in-service performance under cyclic loading and humidity variations to support the development of eco-efficient and resilient flooring systems.

## 1. Introduction

The evaluation of adhesives used in the installation of wooden flooring is a critical aspect of ensuring the durability, safety, and performance of flooring systems [1]. With the increasing diversity of wood species employed in flooring applications, it becomes imperative to assess how different adhesives interact with various wood substrates [2]. This introduction provides an overview of the methodologies utilized to evaluate adhesives for wooden flooring, emphasizing the importance of selecting appropriate testing techniques to ensure optimal adhesion and longevity of flooring installations [3].

Adhesives play a pivotal role in the structural integrity and longevity of wooden flooring systems [4]. The bond between the wood substrate and the adhesive must withstand various stresses, including mechanical loads, thermal fluctuations, and environmental conditions [5]. Inadequate adhesive performance can lead to flooring failures such as delamination, warping, and reduced load-bearing capacity [6]. Therefore, comprehensive evaluation methods are essential to predict and enhance the performance of adhesive bonds in wooden flooring applications [7].

Mechanical tests are fundamental in assessing the strength and durability of adhesive bonds in wooden flooring [8]. These tests simulate real-world forces and provide quantitative data on adhesive performance [9]. Shear strength testing measures the adhesive bond’s ability to resist shear forces, which are common in flooring applications [10]. Studies have utilized double lap shear tests to analyze the influence of adhesive type and quantity on the failure modes and shear strength of timber–concrete joints, providing insights into optimal adhesive application techniques [11]. Pull-off tests evaluate the tensile strength of the adhesive bond perpendicular to the substrate surface [12]. Research has analyzed the pull-off strength between wood flooring and ground surfaces to mitigate construction defects [13]. Lap shear testing assesses the adhesive bond’s resistance to shear forces along the plane of adhesion [14]. Investigations into the influence of wood moisture content during adhesive bonding have employed lap shear testing to understand the effects on bond strength [15].

Assessing the long-term performance of adhesives under various environmental conditions is crucial for predicting their suitability in flooring applications [16]. Aging tests involve exposing specimens to accelerated aging processes, including wetting–drying cycles, freezing–thawing, and exposure to ultraviolet radiation, to evaluate the adhesive bond’s durability [17]. Studies have examined the effects of such conditions on timber–glass bonds, revealing the impact of environmental factors on adhesive performance [18]. Evaluating the adhesive’s performance at elevated temperatures is essential, especially for flooring exposed to underfloor heating systems [19]. Research has investigated the material properties of wood and adhesive bonds at various temperature levels below the char temperature of wood, providing insights into thermal stability [20].

Beyond conventional mechanical tests, advanced analytical methods offer deeper insights into the adhesive bond’s characteristics [21]. Nanoindentation is one such technique that measures the mechanical properties of the adhesive at the microscopic level, providing data on hardness and elastic modulus [22]. It has been employed to study the micromechanics of wood bonded at different moisture contents, elucidating the effects on adhesive performance [23]. The Automated Bonding Evaluation System allows for the assessment of adhesive bond strength development over time, facilitating the study of adhesive reactivity with various substrates [24].

The shift toward sustainable construction practices has led to the development and evaluation of environmentally friendly adhesives [25]. Research has focused on adhesives derived from natural sources, such as tannins, to reduce environmental impact [26]. Studies have explored the use of tannin-based adhesives for engineered flooring, demonstrating their potential as eco-friendly alternatives [27]. Conducting life-cycle assessments of bio-adhesives provides a comprehensive understanding of their environmental footprint from production to application [28]. Such assessments have been conducted to compare bio-adhesives with conventional fossil-based resins, highlighting the benefits and challenges associated with their use [29].

The selection of wood species and adhesive system directly affects the longevity and performance of flooring [30]. Oak (*Quercus robur* L.) is widely recognized for its strength and wear resistance, though it is hygroscopic and may expand or contract with humidity changes [31]. Maple (*Acer platanoides* L.) offers a more uniform texture but is less UV-resistant [32]. Hickory (*Carya* Nutt.) provides extreme hardness but can be difficult to process [33]. Cherry (*Cerasus*) and walnut bring high aesthetic value but are generally softer, which makes them prone to surface damage [34]. Oak exhibits an average density of 720–750 kg/m^3^ and high bending strength (~100 MPa). Teak (*Tectona grandis* L.f.) has a density of ~660 kg/m^3^ with excellent dimensional stability due to its high oil content. Iroko (*Milicia excelsa* (Welw.) C.C. Berg) shows a density of ~690 kg/m^3^ and moderate durability. Wenge (*Millettia laurentii* De Wild.) is very dense (~860 kg/m^3^) with high hardness, making it suitable for heavy-duty flooring. Merbau (*Intsia bijuga* (Colebr.) Kuntze) has a density of ~850 kg/m^3^ and high natural durability. These figures highlight the variability in density and mechanical performance, which directly influences adhesive bonding behavior.

Installation methods vary depending on the substrate and wood type [35]. The glue-down method offers maximum stability for both solid and engineered wood [36]. Floating floors, often used with engineered planks, reduce subfloor preparation time but may exhibit more movement [37]. Nail-down methods are traditional and effective but require wood subfloors and professional tools [38].

Environmental factors such as temperature fluctuations, humidity, UV exposure, and mechanical wear significantly influence flooring longevity [39]. Moisture is particularly critical, as wood expands and contracts, which can compromise adhesive bonds [40]. Proper acclimatization of materials and use of moisture barriers are essential [41]. UV radiation can alter the color and properties of both wood and adhesives, while repeated mechanical stress from foot traffic leads to surface degradation [42].

In conclusion, optimizing wood flooring systems requires integrating knowledge about wood species, adhesive properties, mechanical and environmental testing, and sustainability aspects [43]. As building standards increasingly emphasize eco-friendliness and durability, the role of rigorous adhesive evaluation and appropriate material selection becomes ever more vital [44].

The aim of this study was to evaluate the performance of semi-elastic polyurethane adhesives for bonding flooring elements made of both temperate hardwood (oak) and tropical hardwood species (teak, iroko, wenge, merbau). The research focused on mechanical performance under standard and thermally aged conditions, surface wettability and roughness, and adhesive penetration mechanisms, with the goal of establishing species-specific guidelines for durable flooring applications.

## 2. Materials and Methods

The experimental program aimed to determine the mechanical and surface properties of adhesive joints used in the installation of wooden flooring with polyurethane adhesives. The samples were prepared from a selection of exotic wood species—iroko, teak, and wenge—as well as from merbau and domestic oak, which served as reference materials. The adhesive used was a one-component semi-elastic polyurethane adhesive (SikaBond® T52 FC, Sika AG, Baar, Switzerland). All wooden lamellas were conditioned to 8 ± 1% moisture content prior to bonding, and densities were determined gravimetrically (oak: 730 kg/m^3^, iroko: 690 kg/m^3^, teak: 660 kg/m^3^, wenge: 860 kg/m^3^, merbau: 850 kg/m^3^).

All samples were prepared under controlled laboratory conditions. For each wood species, two types of specimens were prepared: for shear strength and tensile strength evaluation. In the case of shear strength tests, wooden strips were bonded in an overlap configuration to achieve a bonding area of approximately 600 mm^2^. The dimensions of the bonded specimens were (160 ± 5) mm × (23 ± 1) mm × (8.0 ± 0.3) mm, and the bond line thickness exceeded 1 mm. All bonds were formed within the open time recommended by the adhesive manufacturer to ensure optimal curing conditions. Tensile strength specimens consisted of wooden lamellas bonded directly to standardized concrete blocks.

For each test series, at least ten replicate specimens were prepared (*n* ≥ 10) for both shear and tensile strength evaluations. Contact angle measurements were conducted with five droplets per specimen, repeated on five specimens per species (*n* = 25). Surface roughness analysis involved three representative specimens per species, with multiple measurement fields per sample. Scanning electron microscopy (SEM) was performed on at least three specimens per condition and species. Statistical analysis was performed using one-way ANOVA with Tukey’s post hoc comparisons, with significance accepted at *p* < 0.05. Results are presented as mean ± standard deviation, and error bars in figures indicate standard deviation.

Mechanical testing was conducted using a Zwick/Roell Z020 universal testing machine (Ulm, Germany). Artificial aging was carried out in a Binder KBF 240 climate chamber (Tuttlingen, Germany). Surface roughness was measured using a KEYENCE VHX-7000N optical microscope (Osaka, Japan), and SEM imaging was performed using a Carl Zeiss Sigma 500 VP FEG (Oberkochen, Germany).

### 2.1. Shear Strength Testing Methodology

**Figure 1:**
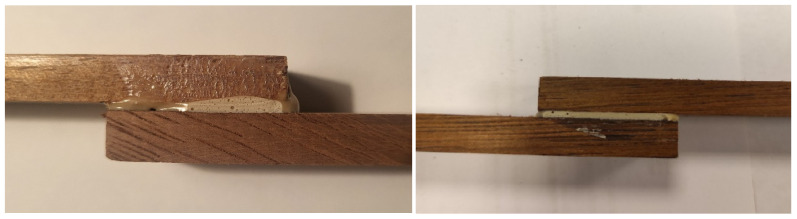
Samples in the merbau–merbau system for strength testing.

The shear strength of adhesive joints was evaluated following the PN-EN ISO 17178 standard [45] (Figure 1), which specifies procedures for testing adhesives used in floor construction. Testing was conducted using a universal testing machine equipped with a constant-rate extension module. Two series of shear tests were performed:

- Series 1: Specimens conditioned in laboratory conditions (23 ± 2 °C, RH 50 ± 5%) for 3 days.
- Series 2: Specimens subjected to artificial aging for 28 days: 7 days in laboratory conditions, 20 days at (40 ± 2)°C, and 1 final day in laboratory conditions.

The testing speed was set to (250 ± 50) N/s in accordance with the standard. The failure modes were recorded to evaluate the integrity and nature of adhesive bonding under different conditioning regimes. Visual inspections of the bonded areas before and after testing allowed additional qualitative assessments.

### 2.2. Tensile Strength Testing Methodology

**Figure 2:**
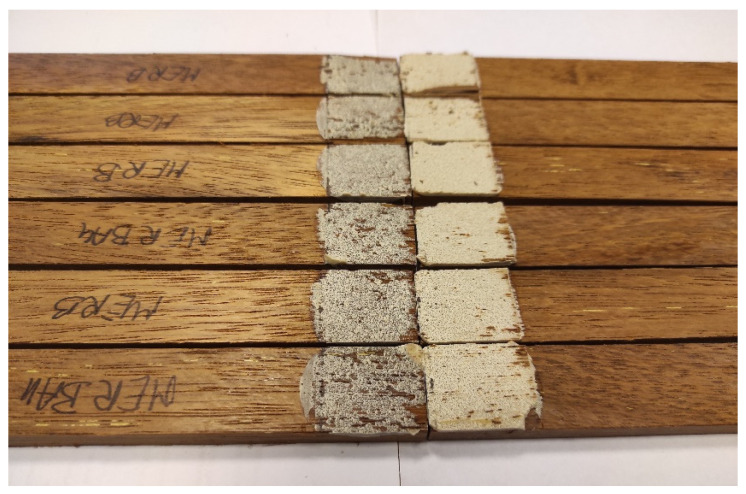
Samples in the merbau–merbau system for tensile testing.

Tensile tests were conducted for wood-to-concrete bonded specimens to assess the adhesive’s ability to resist perpendicular tensile forces (Figure 2). The following testing regimes were implemented:

- Series 1: 7 days in laboratory conditions.
- Series 2: 28 days in laboratory conditions.
- Series 3: 28-day artificial aging cycle identical to shear tests.

The tensile strength was measured using a tensile testing machine fitted with a pull-off attachment designed for composite samples. During testing, failure modes (adhesive, cohesive in adhesive, cohesive in wood, or cohesive in concrete) were identified and documented. These failure modes help diagnose adhesive compatibility and potential substrate weaknesses.

### 2.3. Contact Angle Measurement Methodology (Wetting Properties)

**Figure 3:**
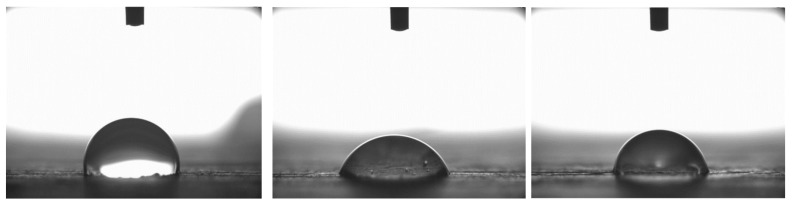
Contact angle measurement setup showing droplets deposited on wood surfaces of oak, iroko, and teak. Each sample represents one of the five tested species.

The wetting properties of the wood surfaces were assessed by measuring the static contact angle of demineralized water droplets using a PG-X goniometer manufactured in the USA. For each measurement, 4 µL droplets (Figure 3) were placed on the wood surface. While droplet behavior was monitored over 60 s, the static contact angle was consistently recorded at 30 s after deposition, as preliminary observations showed that changes beyond this point were minimal (<2° variation), ensuring comparability across species. Five droplets per species were analyzed. Smaller contact angles indicate better wettability, which is crucial for adhesive penetration and bond formation. Measurements were conducted on five wood species: oak, merbau (analyzed previously in 2023), and wenge, iroko, and teak (analyzed in 2024). The contact angle was measured at 10 s after droplet deposition, when the droplet shape had stabilized and no rapid changes in the contact angle were observed. The analysis provided valuable insights into the interaction between the wood surface and the adhesive system, influencing both bond quality and long-term durability.

### 2.4. Surface Roughness Meassurment Methodology 

Surface texture was evaluated using a KEYENCE VHX-7000N digital optical microscope (Osaka, Japan) in collaboration with the Military University of Technology (WAT, Warsaw, Poland). To simulate typical flooring preparation, wood surfaces were sanded with 120-grit paper, and dust was carefully removed using compressed air. High-resolution, non-contact three-dimensional topography scanning was then performed over an area of 1.0 × 1.0 mm. To minimize the influence of natural waviness, scanned regions were aligned to the mid-zone of each lamella, avoiding edges and anatomical irregularities. Surface roughness parameters (Sa, Sz, Sp, Sv, Ssk, Sku) were subsequently calculated using a reference length of 0.8 mm, in accordance with ISO 4287/25178 standards.

The following roughness parameters were measured:

- Sa: Arithmetical mean height of the surface.
- Sz: Maximum height of surface features.
- Sp: Maximum peak height.
- Sv: Maximum valley depth.
- Ssk: Surface skewness (asymmetry).
- Sku: Surface kurtosis (peakedness).

**Figure 4:**
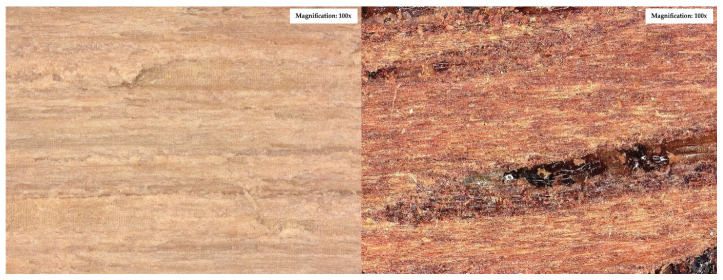
Optical roughness analysis: representative scanned areas of each wood species, showing variations in surface morphology.

Measurements were conducted on the five wood species (Figure 4). These data were used to correlate surface morphology with adhesive performance and to identify how topographical differences influence bond strength.

### 2.5. Scanning Electron Microscopy (SEM)

To evaluate the microstructure of adhesive joints, scanning electron microscopy was performed using a Carl Zeiss Sigma 500 VP SEM (Oberkochen, Germany) with a field emission gun (FEG) for high-resolution imaging. All specimens were gold-sputtered to enhance conductivity prior to imaging. Observations were conducted at accelerating voltages of 5 keV using secondary electron (SE) detection. SEM observations included adhesive cross sections in the wood–adhesive–wood systems for five types of wood under two conditions:

- After laboratory conditioning.
- After thermal aging (7 days lab + 20 days at 40 ± 2 °C + 1 day lab).

**Figure 5:**
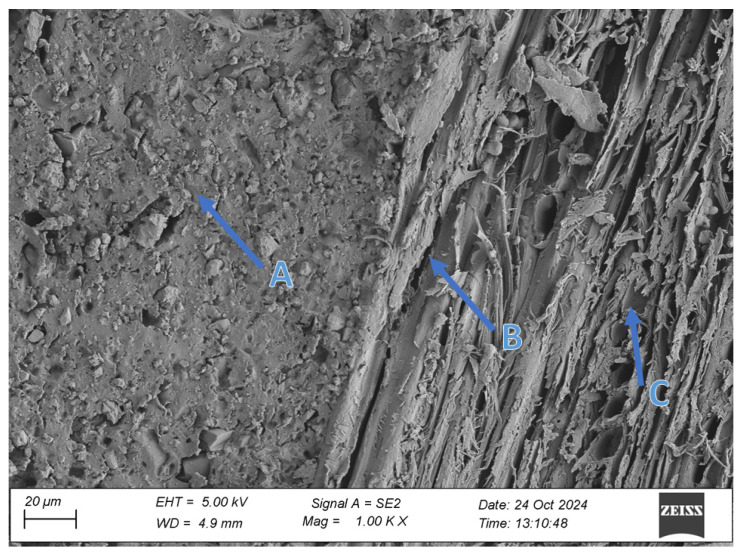
Example SEM image description: (A) adhesive; (B) phase boundary between the adhesive and the wood; (C) wood (in this particular image, oak).

Images were captured at magnifications of 100×, 500×, 1000×, and 2000× to evaluate adhesive penetration, pore formation, and interfacial interactions. The results provided essential data on adhesive behavior at the microscopic level, including insights into porosity, phase boundaries, and structural integrity post-aging. An exemplary description of the SEM images has been provided for the selected micrograph, in this case for oak. The same procedure was applied to all other SEM images (Figure 5).

All tests followed strict quality control protocols based on ITB internal standards and the European PN-EN ISO framework.

## 3. Research Results

### 3.1. Shear Strength Testing (PN-EN ISO 17178)

**Figure 6:**
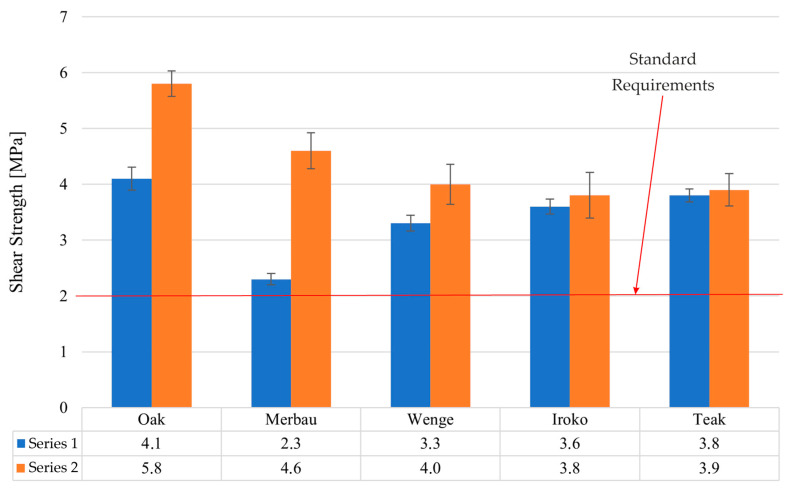
Shear strength test results for all tested samples.

The adhesive joints tested for shear strength demonstrated solid and reproducible bonding characteristics across all analyzed exotic wood species (Figure 6).

In Series 1, representing samples conditioned under laboratory conditions for 3 days, the average shear strength results were as follows: wenge—3.3 MPa, iroko—3.6 MPa, and teak—3.8 MPa. These results indicated that, even without thermal pre-conditioning, the bond quality met and significantly exceeded the minimum threshold of 2 MPa as specified in PN-EN ISO 17178 for semi-elastic adhesives.

After 28 days of artificial aging (Series 2), comprising 7 days of laboratory conditioning, followed by 20 days at (40 ± 2) °C and an additional day under standard conditions, the shear strength values increased in most cases: wenge—4.0 MPa, iroko—3.8 MPa, and teak—3.9 MPa. The percent increase in shear strength after thermal aging was most significant for wenge (21.2%), followed by iroko (5.6%), and teak (2.6%).

These results underscore the excellent performance of the polyurethane adhesive system under elevated temperature exposure. The increase in bond strength observed after thermal conditioning is most likely associated with additional crosslinking and stress relaxation within the adhesive layer, rather than with further penetration into the substrate. SEM micrographs confirmed a stable adhesive–wood interface but did not show evidence of increased penetration after curing. Wenge, despite having the lowest initial shear strength, exhibited the most pronounced strength gain, which suggests delayed stabilization of interfacial adhesion properties. In contrast, teak’s minimal strength increase implies high initial compatibility between the adhesive and substrate. Importantly, none of the wood types demonstrated bond degradation due to thermal exposure, affirming the adhesive’s thermal durability and suitability for flooring applications where long-term mechanical stability is essential.

### 3.2. Tensile Strength Testing (PN-EN ISO 17178)

**Figure 7:**
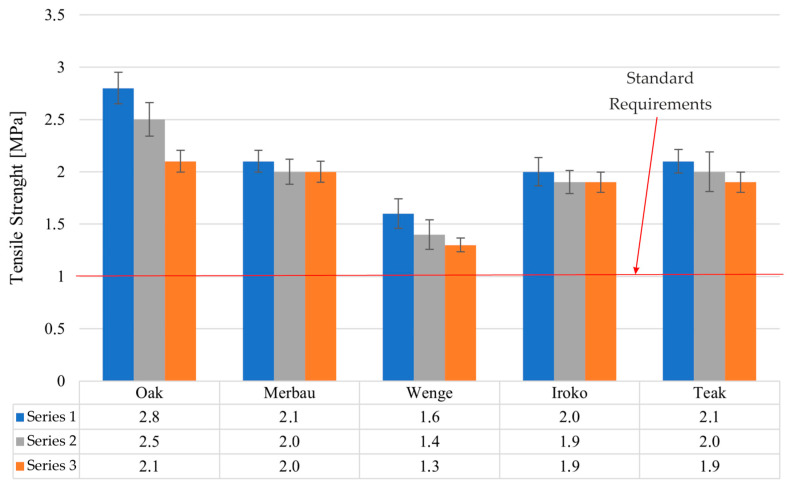
Test results for all tested samples.

Tensile strength evaluation of the adhesive bonds formed between wooden elements and concrete blocks yielded greater inter-species variability than shear strength testing (Figure 7).

In Series 1, the tensile strength values were as follows: teak—2.1 MPa, iroko—2.0 MPa, and wenge—1.6 MPa. This hierarchy suggests better initial compatibility of teak and iroko with the concrete–adhesive interface.

After 28 days of laboratory conditioning (Series 2), tensile strength slightly declined or remained stable: teak—2.0 MPa (−4.8%), iroko—1.9 MPa (−5.0%), and wenge—1.4 MPa (−12.5%). Series 3, which involved thermal aging, showed additional minor reductions: teak—1.9 MPa (−9.5% overall decline), iroko—1.9 MPa (−5.0%), and wenge—1.3 MPa (−18.8%).

Despite these decreases, all tested values remained well above the 1 MPa minimum standard. The moderate decline in tensile strength following thermal exposure may be attributed to moisture redistribution and differential expansion at the wood–concrete interface, which can induce microcracking or stress concentration zones. Teak and iroko demonstrated the most stable performance, with overall declines of less than 10%, suggesting strong compatibility and long-term resilience. Wenge’s bond strength deterioration suggests it is more sensitive to environmental fluctuations, possibly due to its denser, less porous structure, which limits adhesive penetration. Known values of volumetric shrinkage are ~12% for oak, 7% for teak, 8% for iroko, and 9% for wenge, consistent with the observed adhesive performance. Teak’s low water uptake explains its stable tensile performance, while wenge’s higher movement potential likely contributed to the larger reductions in bond strength after aging

### 3.3. Contact Angle Measurement (Wetting Properties)

**Figure 8:**
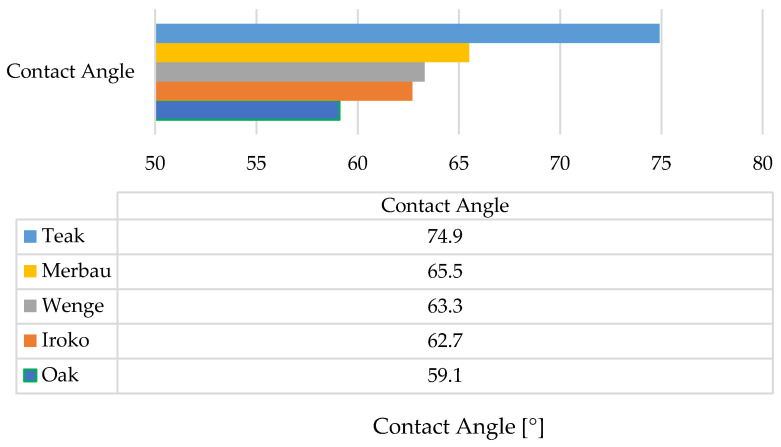
Contact angle measurement results for all tested samples.

The static contact angle measurements of water droplets provided insight into surface wettability, which is a critical factor affecting adhesive spread and penetration (Figure 8).

The lowest contact angle was recorded for oak (59.1°), indicating a hydrophilic surface conducive to adhesive wetting. Among exotic species, iroko showed a value of 62.7°, wenge 64.4°, merbau 65.5°, and teak the highest at 74.9°. Comparatively, teak’s contact angle was 26.8% higher than oak’s, suggesting considerably lower surface energy. Oak (class 2–3, ~5% extractives), teak (class 1, up to 15% oily extractives), iroko (class 1–2, ~7%), wenge (class 1, ~8%), merbau (class 1, ~12%)—these values contextualize differences in bonding behavior and wettability. These findings indicate teak’s known natural resistance to moisture and chemical interaction, likely due to its high content of extractives and surface oils. On the other hand, oak and iroko surfaces exhibited high wettability, which correlates with more effective adhesive bond formation.

The results are consistent with trends observed in mechanical testing: teak’s relatively poor wettability contrasts with its high initial mechanical performance, indicating that factors beyond wettability—such as microstructural interlocking or adhesive compatibility—also play major roles. Teak’s exceptionally high contact angle, in combination with its very rough and textured surface (as revealed in 3D optical microscopy), suggests that mechanical interlocking compensates for limited adhesive wetting. Despite low surface energy, the physical retention of adhesive within deep surface irregularities may promote effective bonding, particularly in shear.

Conversely, wenge’s moderate contact angle (64.4°) suggests an intermediate surface energy—not as hydrophobic as teak, but not as receptive to wetting as oak or iroko. However, in contrast to teak, wenge exhibited the smoothest surface topography and the lowest roughness parameters, which likely limited both adhesive spread and penetration. The combination of moderate wettability and minimal mechanical anchoring may explain the relatively lower tensile strength values recorded for wenge, especially after thermal aging. Nevertheless, wenge still showed a significant increase in shear strength after aging, implying that under certain conditions (e.g., elevated temperature), adhesive flow and polymerization may improve interfacial compatibility, even in substrates with less favorable surface properties.

**Figure 9:**
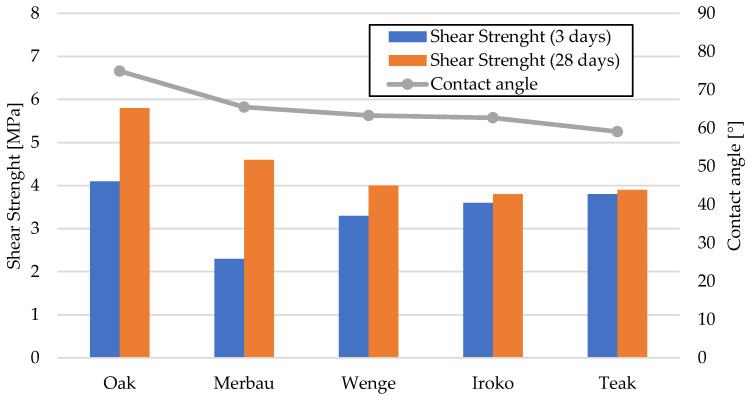
Correlation between average static contact angle and shear strength after laboratory conditioning.

To better visualize the relationship between wettability and bond strength, Figure 9 illustrates the correlation between average static contact angle and shear strength after laboratory conditioning. While oak and iroko, with low contact angles, achieved high shear strength, teak maintained strong bonding despite poor wettability due to mechanical interlocking effects.

### 3.4. Surface Roughness Analysis

**Figure 10:**
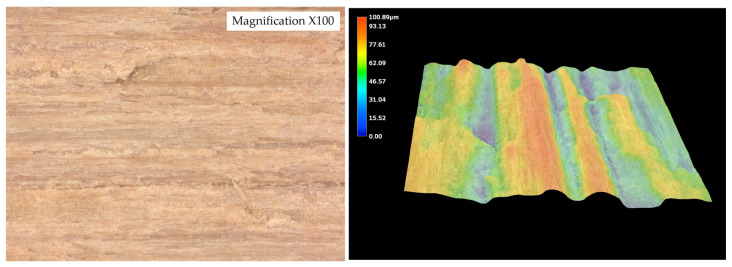
Tested sample surface of oak. Color scale represents surface height (blue = valleys, red = peaks).

**Figure 11:**
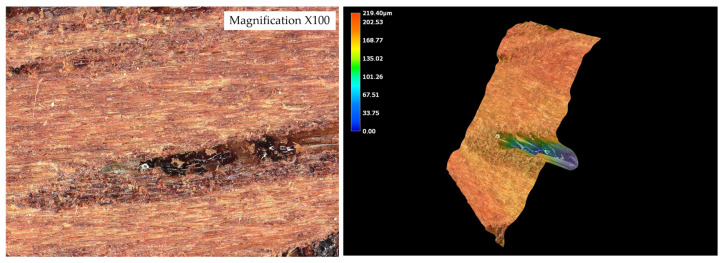
Tested sample surface of merbau. Color scale represents surface height (blue = valleys, red = peaks).

**Figure 12:**
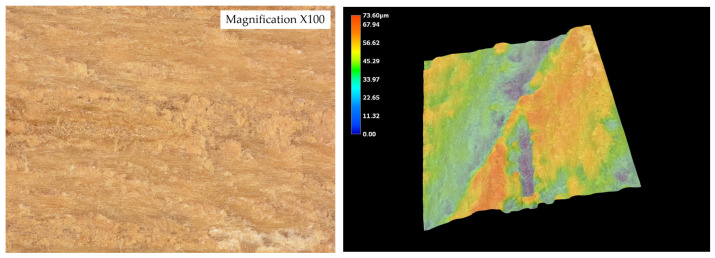
Tested sample surface of iroko. Color scale represents surface height (blue = valleys, red = peaks).

**Figure 13:**
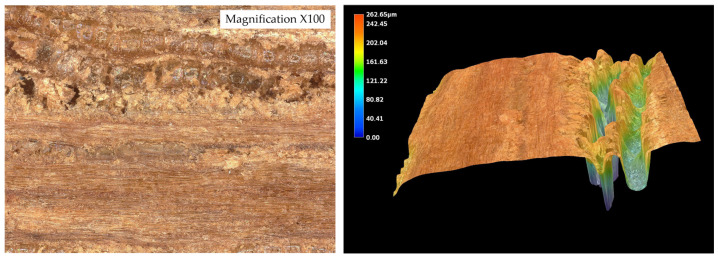
Tested sample surface of teak. Color scale represents surface height (blue = valleys, red = peaks).

**Figure 14:**
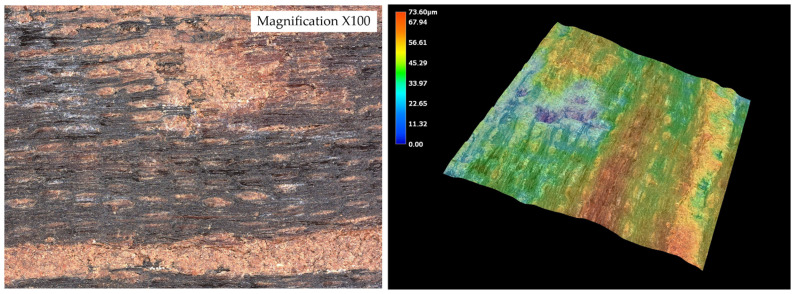
Tested sample surface of wenge. Color scale represents surface height (blue = valleys, red = peaks).

The surface roughness parameters obtained using optical microscopy revealed marked differences in surface topography among the tested wood species (Figure 10, Figure 11, Figure 12, Figure 13 and Figure 14) and (Table 1).

The surface roughness parameters obtained using optical microscopy revealed marked differences in surface topography among the tested wood species (Figure 9, Figure 10, Figure 11, Figure 12 and Figure 13 and Table 1). These topographical features play a critical role in the mechanical anchoring of adhesives and directly influence bond formation quality.

Among all tested species, teak exhibited the highest roughness values, with an arithmetical mean height (Sa) of 25.136 µm and a maximum surface height (Sz) of 221.636 µm. The maximum peak height (Sp) reached 44.366 µm, while the maximum valley depth (Sv) was 177.270 µm. These values point to a surface characterized by deep valleys and sharp peaks, resulting in a highly irregular and textured morphology. Such a profile enhances mechanical interlocking potential by creating microcavities where adhesive can accumulate and anchor upon curing. However, this same irregularity may also hinder adhesive uniformity, potentially leading to uneven bond lines or void formation if not compensated by appropriate application techniques.

In stark contrast, wenge demonstrated the smoothest surface topography, with Sa = 8.92 µm, Sz = 71.250 µm, Sp = 29.672 µm, and Sv = 41.578 µm. These significantly lower values indicate a much flatter and more homogenous surface, with minimal depth and height variation. Such a surface offers limited opportunities for mechanical interlocking and is generally considered less favorable for adhesive anchorage through physical retention mechanisms. Despite this, wenge achieved substantial improvements in shear strength after aging, suggesting that alternative adhesion mechanisms—such as strong cohesive strength within the adhesive layer or interfacial compatibility at the molecular level—played a compensatory role.

The contrast between teak and wenge is particularly striking. Teak’s Sa value was approximately 181.7% higher than that of wenge, while its Sz was more than three times greater. The Sv value for teak was over four times higher than that of wenge, indicating much deeper valleys that could serve as adhesive reservoirs. These substantial differences in surface architecture directly contribute to differing bonding behaviors. Teak’s rugged topography allows for effective adhesive keying even in the presence of hydrophobic extractives that inhibit wetting. This is supported by SEM analysis, which showed limited adhesive penetration into teak despite its high roughness—implying that bonding relies heavily on physical anchoring rather than chemical interaction.

Conversely, wenge’s flat surface—with relatively low Sa and Sz values—provides minimal microstructural anchoring potential. Nevertheless, SEM observations confirmed continuous adhesive layers without delamination, and mechanical testing revealed a notable post-aging increase in shear strength. This suggests that wenge, despite its smooth surface and limited roughness parameters, is capable of forming durable bonds, potentially due to favorable interactions at the adhesive–wood interface or more uniform adhesive distribution that avoids stress concentrations.

While oak, merbau, and iroko occupied intermediate positions in terms of surface roughness—with Sa values of 12.932 µm, 15.666 µm, and 15.366 µm, respectively—the behaviors observed in teak and wenge serve as extreme reference points illustrating the complexity of wood–adhesive interactions. Importantly, the correlation between surface roughness and adhesive performance is nonlinear. High roughness, as in teak, does not guarantee superior tensile strength if adhesive wetting is insufficient, while low roughness, as in wenge, does not preclude strong bonding if other compatibility factors are favorable.

These findings underscore that surface roughness, while a key parameter, must be evaluated in conjunction with other surface characteristics—such as wettability, porosity, and extractive content—as well as adhesive properties like viscosity, curing behavior, and flexibility. The case of teak demonstrates the importance of mechanical interlocking in rough, extractive-rich species, whereas wenge highlights the adhesive’s ability to adapt and perform even in conditions of minimal physical anchoring.

**Figure 15:**
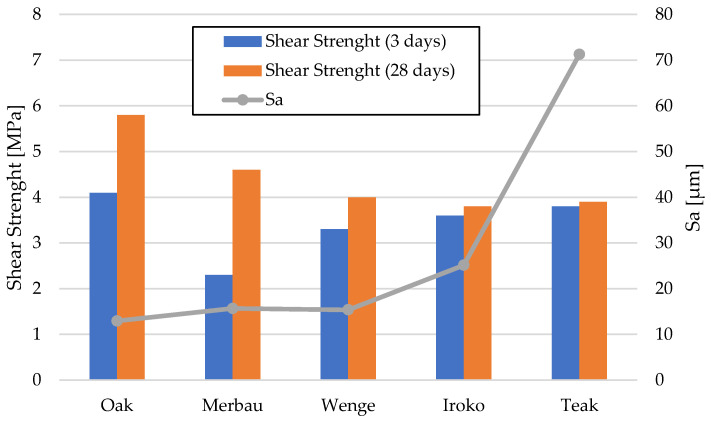
Correlation between surface roughness (Sa) and shear strength of given wood specimens.

Figure 15 illustrates the nonlinear relationship between surface roughness (Sa) and shear strength. While teak exhibited the highest roughness and strong bonding, wenge, with very low roughness, also achieved significant strength gains after aging. This confirms that adhesive performance is governed not by roughness alone but by the interplay of surface morphology, extractives, and adhesive curing behavior.

### 3.5. Microstructure Analysis

SEM imaging revealed that the adhesive layer in both the oak and merbau joints maintained integrity regardless of the conditioning method (Figure 16, Figure 17, Figure 18, Figure 19, Figure 20, Figure 21, Figure 22, Figure 23, Figure 24 and Figure 25).

SEM imaging revealed that the adhesive layer in all tested wood species—oak, merbau, teak, wenge, and iroko—generally maintained structural integrity regardless of conditioning method. Cross-sectional micrographs at magnifications ranging from 100× to 2000× consistently showed continuous adhesive layers with no signs of interfacial voids or microcracks. The polyurethane adhesive demonstrated a microporous internal structure composed of closed, well-dispersed pores, which remained stable even after thermal aging.

In oak specimens, the adhesive formed a well-developed and uniform interface, with partial penetration into vessel lumens and intercellular spaces. No delamination was observed in any oak samples, confirming strong interfacial adhesion and compatibility between the polyurethane system and the hygroscopic oak structure.

Merbau joints also exhibited continuous adhesive layers in most cases; however, isolated microstructural discontinuities were detected in selected aged samples. These localized detachment zones—manifesting as micro-gaps at the wood–adhesive interface—suggest the presence of adhesive instability potentially linked to merbau’s dense structure and high content of natural extractives. These extractives may have interfered with wetting or bonding at the microscopic level. Despite these sporadic delaminations, adhesive penetration into the merbau tissue was generally observed, particularly in thermally aged specimens, indicating that elevated temperature may facilitate deeper penetration by increasing adhesive fluidity during early curing. While the majority of samples retained cohesive bond integrity, the presence of interface irregularities highlights the need for tailored bonding strategies when working with chemically active tropical hardwoods like merbau.

In teak samples, SEM imaging revealed a continuous and undamaged adhesive layer under both laboratory and aged conditions. Although adhesive penetration into teak’s structure was more superficial—likely due to its high oil content and low surface energy—the bonds remained morphologically stable. The combination of high surface roughness (as confirmed by optical profilometry) and microstructural irregularities appeared to support mechanical interlocking, compensating for poor wettability. Aged teak specimens exhibited slightly deeper adhesive infiltration, reinforcing the notion that thermal exposure enhances initial bond formation, even in extractive-rich woods. The absence of delamination or microcracking confirms the effectiveness of polyurethane adhesives for bonding teak under elevated temperatures.

Wenge specimens exhibited sharply defined and structurally intact adhesive interfaces, with limited penetration into the wood matrix. This was expected, given wenge’s very dense and low-porosity anatomical structure. Despite moderate surface wettability and the lowest surface roughness among all tested species, no interfacial voids or delamination zones were identified. Interestingly, aged wenge samples showed substantial mechanical strength gains, suggesting that chemical bonding or improved polymer crosslinking may play a larger role than mechanical anchoring in this case. SEM observations confirmed the adhesive’s dimensional compatibility with the substrate and validated its long-term resilience in challenging bonding scenarios.

In iroko samples, SEM analysis showed consistent and continuous bonding lines, characterized by gradual phase transitions and well-developed interfacial contact. Adhesive penetration into vessel lumens and ray cells was evident, both before and after thermal aging. Iroko’s favorable surface characteristics—moderate roughness and high wettability—enabled thorough adhesive wetting and stable interface formation. Aged specimens displayed slightly increased penetration depth, further confirming the beneficial role of elevated temperature in enhancing adhesive diffusion. No interfacial degradation or delamination was observed, corroborating iroko’s excellent compatibility with polyurethane adhesives.

## 4. Discussion

The results obtained in this study confirm that the polyurethane adhesive used for bonding wooden flooring provides robust mechanical properties and reliable performance under variable environmental conditions. These findings are in agreement with other scientific works focusing on adhesive bonding of hardwoods and exotic species.

The increase in shear strength after thermal aging observed in all tested wood species, especially in wenge (a 21.2% improvement), confirms the adhesive’s ability to maintain or even enhance bond integrity when subjected to elevated temperatures. This phenomenon may be attributed to continued polymerization of the adhesive or improved diffusion into the wood microstructure during the aging cycle. These findings are consistent with results reported by Stoeckel et al. [1]. Similarly, Frihart and Hunt confirmed that semi-elastic polyurethane adhesives exhibit resilience and thermal adaptability suitable for long-term floor applications [4].

Despite some minor reduction in tensile strength values after thermal conditioning (up to 18.8% in wenge), all specimens remained above the required 1 MPa standard. The slight decrease can be explained by the development of internal stress at the wood–concrete interface caused by differential expansion. It is worth noting that teak and iroko maintained stable performance, indicating that their internal structures and interaction with polyurethane adhesives are less susceptible to environmental fluctuation. These observations correspond with findings from Kamke and Sinha [8,9].

The wood–concrete bonding system exhibited greater variability than wood–wood joints. Tensile strength values remained above the 1 MPa requirement, but species-specific differences were observed. Teak and iroko demonstrated stable performance after aging (declines <10%), while wenge showed larger reductions (up to 18.8%). This suggests that dense, low-porosity substrates like wenge are more sensitive to differential shrinkage and stress concentration at the wood–concrete interface. Practical implications include the use of primers or moisture barriers when installing tropical hardwood flooring on mineral substrates to ensure long-term adhesion stability.

The wettability results align with known characteristics of the wood species studied. Teak exhibited the highest contact angle (74.9°), suggesting a more hydrophobic surface likely due to its natural oil content, as described by Yeh et al. [20]. Despite its relatively poor wettability, teak showed high mechanical performance, which suggests that bonding quality is influenced not only by surface energy but also by surface morphology and microstructure compatibility. On the contrary, oak exhibited the lowest contact angle and high bonding performance, confirming the general trend that high wettability promotes better adhesion, in line with results from Kamke and Lee [7].

Surface roughness measurements revealed notable trend between Sa values and adhesive performance. Teak had the highest Sa and Sz parameters, potentially allowing for enhanced mechanical interlocking of the adhesive. These results align with those of Söğütlü and Sudol [38,46]. However, wenge, with the lowest roughness, demonstrated strong initial bonding, showing that other factors such as microstructure, porosity, and extractive content must also be considered. The inconsistencies between surface roughness and tensile strength further support the hypothesis presented by Tran et al. [21]. An optimal range of surface roughness for polyurethane bonding appears to be Sa = 12–18 µm, as observed for oak, iroko, and merbau. Below this threshold (wenge), limited interlocking reduces performance, while excessively high values (teak) may cause adhesive pooling and stress concentration. Our findings align with Seers [22], who observed reduced penetration in dense hardwoods, and differ from Stadlmann et al. [23], who reported stronger influence of surface wettability alone. By integrating roughness, wettability, and mechanical testing, our study provides a more comprehensive framework.

SEM imaging showed a well-distributed, microporous adhesive layer with no delamination or cracks after thermal aging, which is an essential indicator of long-term reliability. The adhesive penetration into the wood’s anatomical structures was apparent, particularly in aged samples. This observation is in line with prior findings by Zhang et al. [9]. The consistent morphology between aged and unaged specimens supports the idea that polyurethane adhesives are highly suitable for dynamic flooring conditions where temperature and humidity can vary seasonally.

This study advances adhesive research beyond conventional single-species evaluations by integrating mechanical, morphological, and microstructural analyses across five wood species. The comparative framework revealed species-specific bonding mechanisms not previously highlighted, such as the role of teak’s pronounced roughness in compensating for poor wettability, or wenge’s delayed adhesion stabilization after thermal exposure. These insights provide a deeper understanding of adhesive–substrate interactions in exotic hardwoods.

From a practical perspective, the results translate into clear recommendations for flooring installation: oak and iroko are well-suited to standard polyurethane application; teak requires slightly thicker adhesive layers to counteract its hydrophobicity; wenge benefits from surface pre-treatment to improve penetration; and merbau requires careful surface preparation to mitigate the effects of extractives. By linking laboratory findings with application strategies, the study enhances both scientific novelty and real-world relevance.

When comparing the present study to commercially available adhesive systems, such as commercial glue, our polyurethane adhesive showed similar levels of performance under both mechanical and environmental stress. Prior product testing by the manufacturer and independent studies have also demonstrated the high resilience of these systems in engineered wood flooring [10].

Lastly, wood species selection is fundamental for bonding effectiveness. Iroko, teak, and merbau showed excellent adhesion results, consistent with their known density and low movement characteristics reported in international wood databases and technical sources [11]. However, it is worth noting that extractives in woods such as wenge may interfere with the polymerization or surface compatibility of adhesives. These factors are well-documented by Pizzi and Rowell [13,31].

Taken together, the findings of this study contribute to a growing body of knowledge supporting the use of polyurethane adhesives in wooden flooring applications, particularly when combined with thermally stable and dimensionally consistent hardwood species. The results validate polyurethane systems as high-performance bonding solutions, suitable for both domestic and exotic woods exposed to varying environmental conditions. A summary of the studies is presented in Table 2.

## 5. Conclusions

This study provides a comprehensive evaluation of polyurethane adhesives used in bonding various wood species for flooring applications, including both domestic (oak) and exotic (iroko, teak, wenge, merbau) hardwoods. Based on a series of mechanical, morphological, and surface analyses, several important conclusions can be drawn.

Firstly, the polyurethane adhesive exhibited excellent shear and tensile strength values across all tested species. All bonded joints exceeded the minimum thresholds defined in the PN-EN ISO 17178 standard, both in their initial state and after thermal aging. Notably, shear strength improved after accelerated aging—especially in wenge wood—indicating continued adhesive curing and compatibility under elevated temperature conditions.

Secondly, tensile strength between wood and concrete showed minor reductions after prolonged aging but remained above critical limits. Teak and iroko, in particular, demonstrated mechanical stability, supporting their use in challenging environments such as radiant floor heating systems or humid interiors.

Contact angle analysis revealed that all tested species maintained wettability sufficient for reliable adhesion. However, variations in hydrophobicity, especially in teak, confirmed that surface chemistry and extractive content must be considered when predicting adhesive performance. Despite higher contact angles, teak achieved robust bonding, which points to the importance of complementary factors such as surface roughness and porosity.

Surface topography data obtained via 3D optical microscopy showed that increased roughness tends to enhance mechanical interlocking, but is not solely predictive of bond quality. The discrepancies between roughness and mechanical outcomes highlight the complex interaction between surface morphology and adhesive flow and curing behavior.

SEM investigations validated the adhesive’s structural integrity at the microlevel. Continuous bonding interfaces, absence of voids or delamination, and successful adhesive penetration across all wood species further support the long-term viability of the tested adhesive system.

Taken together, the research confirms that semi-elastic polyurethane adhesives are well-suited for the installation of engineered and solid wood flooring across a diverse range of hardwood species. The results emphasize the importance of considering wood anatomy, surface properties, and adhesive compatibility when designing flooring systems for long-term performance and environmental resilience.

This work contributes valuable insights into the selection of adhesive systems and wood materials in construction and renovation sectors, supporting the development of durable, eco-effective flooring solutions for residential and commercial applications.

Beyond confirming the suitability of semi-elastic polyurethane adhesives, our results should be interpreted in the wider context of sustainability. Bio-based adhesive systems, including tannin- and lignin-derived polyurethanes, have demonstrated mechanical performance comparable to fossil-based PU while offering reduced environmental impacts. Recent life-cycle assessments indicate that partial substitution of fossil-derived polyols with renewable feedstocks may reduce the carbon footprint and greenhouse gas emissions by 30–50%. In addition, soy protein and bio-epoxy formulations are being optimized for higher water resistance, further broadening the portfolio of sustainable alternatives. Moreover, upcoming EU regulations and market expectations (e.g., Environmental Product Declarations) increasingly reward eco-efficient solutions, underscoring the relevance of this research direction. Consequently, future studies should not only assess the compatibility of such bio-based adhesives with demanding hardwoods like teak and wenge, but also evaluate long-term in-service durability under fluctuating humidity and thermal loads. Such efforts would bridge the gap between laboratory validation and market adoption, supporting the flooring industry’s transition toward circular and environmentally responsible construction materials.

The key conclusions drawn from this study are:

- Polyurethane adhesives provide strong and thermally stable bonding with both domestic and exotic wood species.
- Teak and iroko demonstrate particularly favorable performance in both mechanical and surface-based evaluations.
- Surface roughness and wettability are critical parameters influencing adhesive behavior, though their effects are species-dependent.
- SEM analysis confirms uniform adhesive penetration and durability, even under thermal aging.
- Proper wood species selection, combined with compatible adhesive systems, is essential for ensuring the long-term reliability of bonded flooring structures.

## Figures and Tables

**Figure 16:**
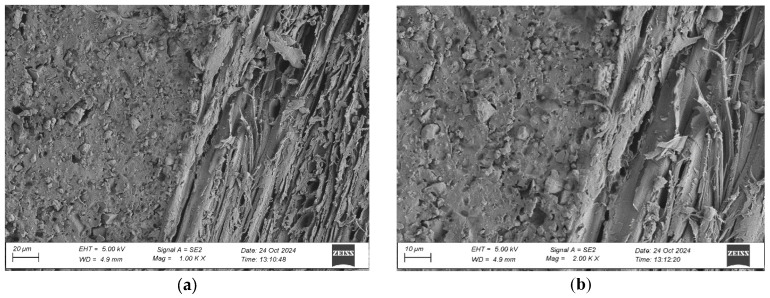
Microstructure of the cross section of the joint in the oak–adhesive joint–oak system after conditioning in laboratory conditions, at magnification: (**a**) 1000× and (**b**) 2000×.

**Figure 17:**
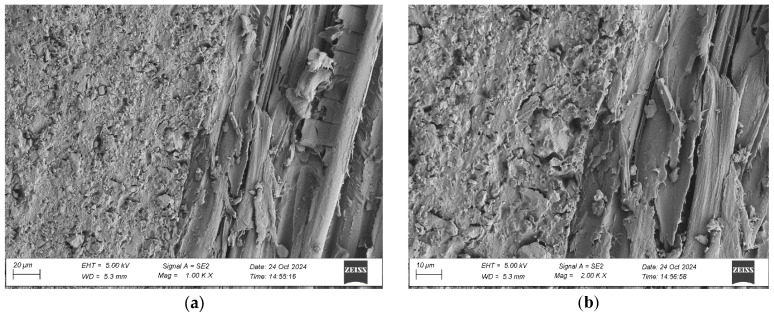
Microstructure of the cross section of the joint in the oak–adhesive joint–oak system after thermal aging, at magnification: (**a**) 1000× and (**b**) 2000×.

**Figure 18:**
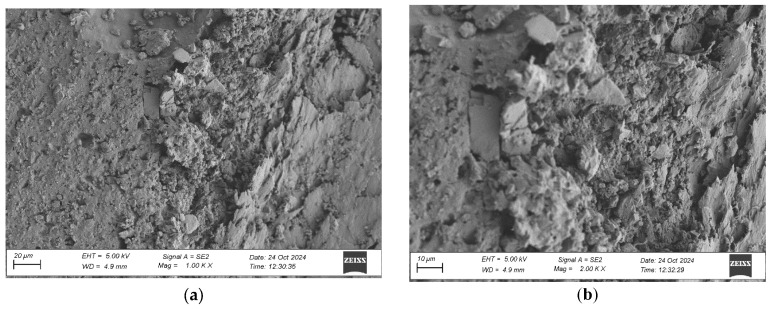
Microstructure of the cross section of the joint in the merbau–adhesive joint–merbau system after conditioning in laboratory conditions, at magnification: (**a**) 1000× and (**b**) 2000×.

**Figure 19:**
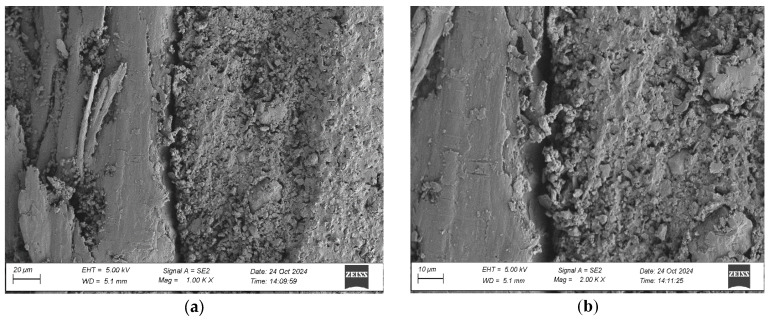
Microstructure of the cross section of the joint in the merbau–adhesive joint–merbau system after thermal aging, at magnification: (**a**) 1000× and (**b**) 2000×.

**Figure 20:**
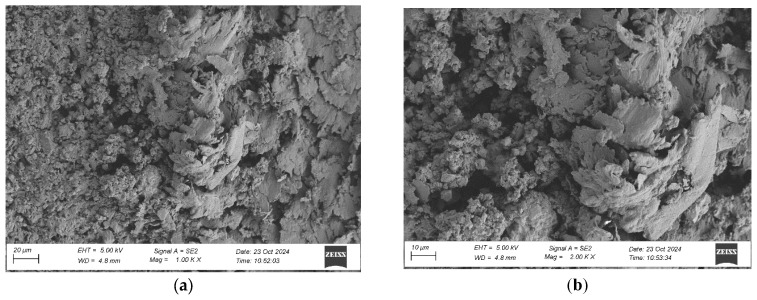
Microstructure of the cross section of the joint in the iroko–adhesive joint–iroko system after conditioning in laboratory conditions, at magnification: (**a**) 1000× and (**b**) 2000×.

**Figure 21:**
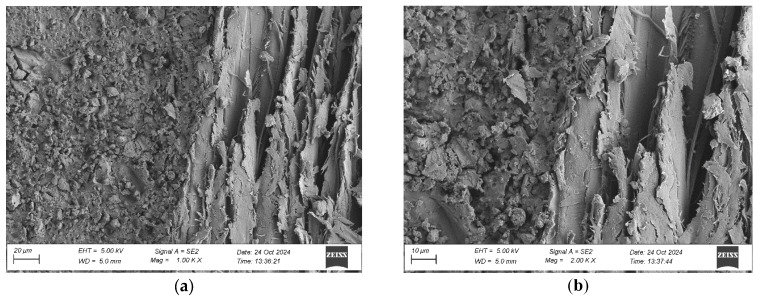
Microstructure of the cross section of the joint in the iroko–adhesive joint–iroko system after thermal aging, at magnification: (**a**) 1000× and (**b**) 2000×.

**Figure 22:**
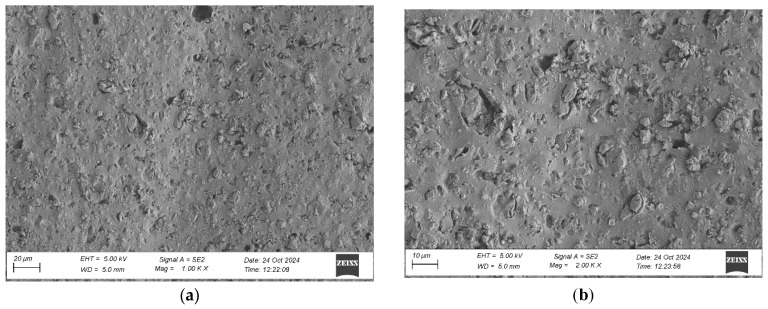
Microstructure of the cross section of the joint in the teak–adhesive joint–teak system after conditioning in laboratory conditions, at magnification: (**a**) 1000× and (**b**) 2000×.

**Figure 23:**
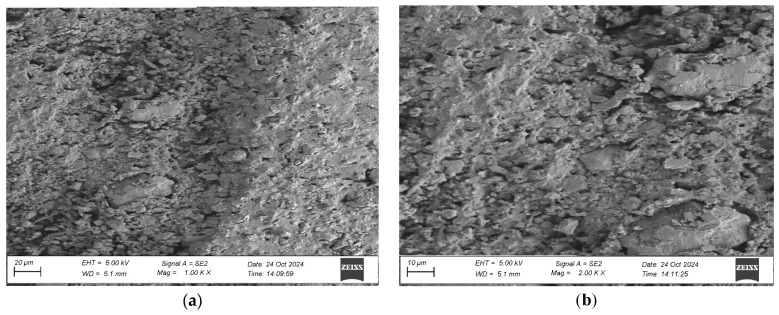
Microstructure of the cross section of the joint in the teak–adhesive joint–teak system after thermal aging, at magnification: (**a**) 1000× and (**b**) 2000×.

**Figure 24:**
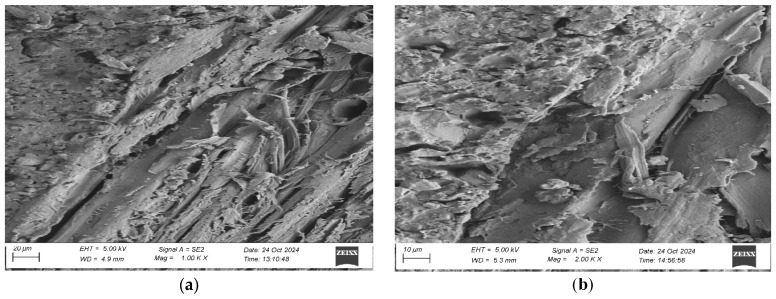
Microstructure of the cross section of the joint in the wenge–adhesive joint–wenge system after conditioning in laboratory conditions, at magnification: (**a**) 1000× and (**b**) 2000×.

**Figure 25:**
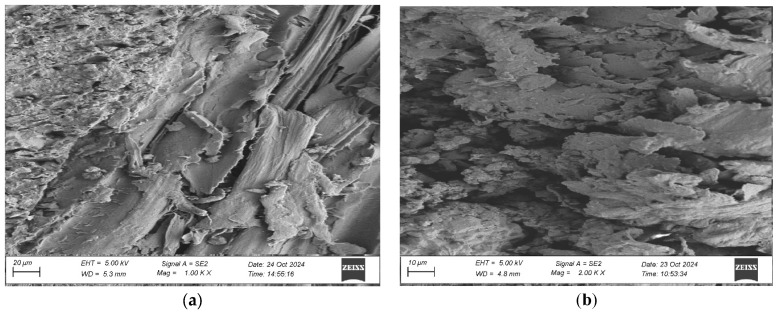
Microstructure of the cross section of the joint in the wenge–adhesive joint–wenge system after thermal aging, at magnification: (**a**) 1000× and (**b**) 2000×.

**Table 1:** Compared results for all tested samples.

Wood Sample	Parameters	Results	Unit
**Oak**	S_a_	12.932	µm
	S_z_	121.838	µm
	S_p_	50.682	µm
	S_v_	71.156	µm
**Merbau**	S_a_	15.666	µm
	S_z_	169.684	µm
	S_p_	31.418	µm
	S_v_	138.266	µm
**Iroko**	S_a_	15.366	µm
	S_z_	131.54	µm
	S_p_	48.716	µm
	S_v_	82.826	µm
**Teak**	S_a_	25.136	µm
	S_z_	221.636	µm
	S_p_	44.366	µm
	S_v_	177.27	µm
**Wenge**	S_a_	8.92	µm
	S_z_	71.25	µm
	S_p_	29.672	µm
	S_v_	41.578	µm

**Table 2:** Comparative summary of wood species properties and adhesive performance.

Species	Density (kg/m^3^)	Contact Angle (°)	Sa (µm)	Shear Strength (MPa)	Tensile Strength (MPa)	Durability Class	Extractives (%)
Oak (*Q. robur*)	730	59.1	12.9	4.1–5.8	2.7–3.3	2–3	~5
Teak (*T. grandis*)	660	74.9	25.1	3.8–3.9	1.9–2.1	1	~15
Iroko (*M. excelsa*)	690	62.7	15.3	3.6–3.8	1.9–2.0	1–2	~7
Wenge (*M. laurentii*)	860	64.4	8.9	3.3–4.0	1.3–1.6	1	~8
Merbau (*I. bijuga*)	850	65.5	15.6	2.3–4.6	2.5–3.0	1	~12

## Data Availability

The original contributions presented in this study are included in the article. Further inquiries can be directed to the corresponding author.
